# A spatiotemporal study of gliosis in relation to depth electrode tracks in drug-resistant epilepsy

**DOI:** 10.1111/ejn.12548

**Published:** 2014-03-26

**Authors:** Joanna Goc, Joan Y W Liu, Sanjay M Sisodiya, Maria Thom

**Affiliations:** 1 Department of Clinical and Experimental Epilepsy, UCL, Institute of NeurologyQueen Square, London, WC1N 3BG, UK

**Keywords:** cortical injury, epilepsy, gliosis, nestin-expressing cells

## Abstract

Key questions remain regarding the processes governing gliogenesis following central nervous system injury that are critical to understanding both beneficial brain repair mechanisms and any long-term detrimental effects, including increased risk of seizures. We have used cortical injury produced by intracranial electrodes (ICEs) to study the time-course and localization of gliosis and gliogenesis in surgically resected human brain tissue. Seventeen cases with ICE injuries of 4–301 days age were selected. Double-labelled immunolabelling using a proliferative cell marker (MCM2), markers of fate-specific transcriptional factors (PAX6, SOX2), a microglial marker (IBA1) and glial markers (nestin, GFAP) was quantified in three regions: zone 1 (immediate vicinity: 0–350 μm), zone 2 (350–700 μm) and zone 3 (remote ≥2000 μm) in relation to the ICE injury site. Microglial/macrophage cell densities peaked at 28–30 days post-injury (dpi) with a significant decline in proliferating microglia with dpi in all zones. Nestin-expressing cells (NECs) were concentrated in zones 1 and 2, showed the highest regenerative capacity (MCM2 and PAX6 co-expression) and were intimately associated with capillaries within the organizing injury cavity. There was a significant decline in nestin/MCM2 co-expressing cells with dpi in zones 1 and 2. Nestin-positive fibres remained in the chronic scar, and NECs with neuronal morphology were noted in older injuries. GFAP-expressing glia were more evenly distributed between zones, with no significant decline in density or proliferative capacity with dpi. Colocalization between nestin and GFAP in zone 1 glial cells decreased with increasing dpi. In conclusion, NECs at acute injury sites are a proliferative, transient cell population with capacity for maturation into astrocytes with possible neuronal differentiation observed in older injuries.

## Introduction

Gliosis, which is synonymous with hypertrophy and/or proliferation of astrocytes, is a common tissue reaction to a variety of central nervous system (CNS) insults from trauma to neurodegeneration (Sofroniew & Vinters, 2010; Kang & Hebert, 2011). In epilepsy, diffuse astrocytic gliosis can be present in the absence of neuronal loss, and is the main neuropathological abnormality in up to 26% of cases in surgical epilepsy series (Love *et al*., 2008). Widespread gliosis is also a frequent observation in post-mortem epilepsy series (Blanc *et al*., 2011). Recuperative benefits of localized glial scar formation following a localized injury include delimitation of the injury site and the reconstitution of the blood–brain barrier (Kang & Hebert, 2011). There is also evidence of neurogenesis as a potential reparative phenomenon following cerebral injury with gliosis (Buffo *et al*., 2008; Boda & Buffo, 2010; Nakayama *et al*., 2010; Hendrickson *et al*., 2011). Long-term detrimental consequences of glial scar formation after injury include hindrance of axonal regrowth and impaired restoration of normal connections and function (Boda & Buffo, 2010). The effects of local glial scar formation on epileptogenesis are less well explored. There has been a recent drift away from a ‘neurocentric’ focus with more directed investigations addressing the participation of astrocytes in the causation of seizures in lesional epilepsies (Steinhauser *et al*., 2012). Astrocytic functions in the CNS are diverse (Chaboub & Deneen, 2012) and extend beyond the mere provision of a supportive scaffold, including roles in potassium buffering, glutamate clearance, blood flow regulation and synchronization of neuronal firing. Dysfunction of any of these processes may be relevant to epileptogenesis (Steinhauser *et al*., 2012). Understanding the mechanisms regulating gliosis in human tissues, in relation to both localized injuries and more widespread gliosis, is therefore of importance.

The source of proliferating astrocytes in the process of gliosis, following either a localized injury or a more generalized process (Wachter *et al*., 2010), is still uncertain. Possibilities include de-differentiation and proliferation of mature and quiescent astrocytes or recruitment and differentiation of progenitor cell types. Mechanisms triggering gliosis include transient loss of blood–brain barrier integrity and up-regulation of pro-inflammatory signals, both of which may occur in association with seizures and epilepsy (Vezzani *et al*., 2011; Heinemann *et al*., 2012; Kovacs *et al*., 2012). Experimental cortical injury models from zebrafish (Baumgart *et al*., 2012) to complex mammals (Frisen *et al*., 1995; Shibuya *et al*., 2002; Nakayama *et al*., 2010) have provided important dynamic systems in which to address questions regarding the spatiotemporal regulation of gliosis in relation to defined cortical injuries. However, animal models, with their reduced complexity, number and range of types of glia, cannot replicate all aspects of astrocytosis in human diseases (Oberheim *et al*., 2009).

A small proportion of patients with drug-resistant epilepsy undergo invasive electroencephalography recording (intracerebral depth electrodes or subdural grid placement), to more accurately localize the seizure onset zone prior to resective surgery (Yuan *et al*., 2012). The interval between electrode placement and tissue resection varies, and injuries of differing stages of cellular reorganization can be identified in tissue sections (Liu *et al*., 2012). These small, focal cortical injuries, of precise ages therefore provide a unique and fortuitous opportunity to study the processes of gliosis and repair in human tissues. Our aim was to study the time course of gliogenesis in relation to electrode track injury sites of varying age in adult patients with epilepsy.

## Materials and methods

### Case selection

Cases were selected from the Epilepsy Tissue Bank at UCL, Institute of Neurology, which has National Research Ethics Committee approval (12/SC/0669); participating individuals signed written consent forms to participate in this study. This study conforms with the code of ethics of the World Medical Association (Declaration of Helsinki). All patients had undergone resective surgery at the National Hospital for Neurology and Neurosurgery for the treatment of drug-resistant epilepsy. The cases selected had additionally undergone previous invasive intracranial monitoring (depth electrode insertion and/or subdural grid placement) at an interval prior to resective surgery and had a focal injury site [henceforth referred to as intracranial electrode (ICE) injuries] identified in the tissue sections. The clinical details of the cases, and the intervals in days from electrode/grid insertion, their removal and tissue resection, are presented in Table1. For the purposes of this study the age of the ICE injury was taken as the time from initial insertion of the intracranial recording device to tissue resection.

**Table 1:** Cases selected for study and clinical and pathology details

Group	Case	Age at resection/gender	Age of ICE track (days)*	Location	Underlying pathology	Nature of injury	Localization of ICE	ICE injury intra-lesional	Complications after ICE recordings
Acute ice injuries	1	25/M	4 [0]	Temporal	FCDIIB	ET	Cortex/WM junction	Yes	None
	2	30/F	8 [0]	Temporal	EFS	SG	Superficial cortex	No	None
	3	18/F	8 [0]	Fronto-parietal	FCDIIA	ET	Cortex/WM junction	No	Slight slowing of fine finger movements at 2 months, pain at operative site
	4	32/F	8 [0]	Parietal	FCDIIB	SG	Superficial cortex	No	None
Subacute ice injuries	5	23/F	10 [0]	Temporal	NSP	ET	Intracortical	No	None
	6	24/F	10 [0]	Temporal	FCDIIB	SG	Superficial cortex	No	Itching at site of implanted electrodes
	7	34/F	10 [0]	Frontal	FCDIIB	ET	Intracortical	Yes	Soft tissue collection drained
	8	40/M	11 [10]	Frontal	FCDIIB	SG	Superficial cortex	No	Initial headache
	9	35/M	13 [6]	Occipital	No pathology (previous DNT)	ET	WM	No	None
	10	29/M	13 [2]	Frontal	FCDIIB	SG	Superficial cortex	No	Expressive dysphasia; resolved
Intermediate ice injuries	11	31/F	28 [17]	Frontal	FCDIIB	SG	Superficial cortex	No	Wound infection, given antibiotics
	12	22/F	30 [21]	Temporal	EFS	ET	CA1 of hippocampus	No	None
	13	25/M	59 [26]	Occipital	NSP	ET	WM	No	Pseudomeningocele
	14	35/M	70 [55]	Temporal	EFS	ET	WM	No	Subgaleal collection
Chronic ice injuries	15	52/M	209 [201]	Frontal	FCDIIB	ET	WM	No	Wound infection, given antibiotics
	16	53/F	232 [222]	Frontal	Normal (adj. to DNT)	ET	Cortex	No	Severe persistent headache
	17	34/M	301 [UK]	Temporal	HS and old contusions	ET	WM	No	None

ET, electrode track; SG, superficial grid lesion; WM, white matter; FCD, focal cortical dysplasia type IIA or B; DNT, dysembryoplastic neuroepithelial tumour; EFS, end folium hippocampal sclerosis; HS, hippocampal sclerosis; NSP, no specific pathology lesion; UK, unknown.

* Age of intracranial electrode track (ICE) is the time interval between the surgical insertion of the electrode and the surgical resection, measured in days; value in brackets is the days between removal of electrode and tissue resection.

### Immunolabelling

Sections were cut from formalin-fixed paraffin-embedded tissue blocks at 5 μm and haematoxylin and eosin (H&E) staining was performed to confirm the location of the ICE lesion. For double-labelled immunohistochemistry, sections were dewaxed, immersed in 3% hydrogen peroxide and de-ionized water for 15 min, microwaved at 800 W for 15 min, cooled and washed in phosphate buffer solution. The first set of primary antibodies, the proliferative cell marker (mini chromosome maintenance protein, MCM2, 1 : 900; BD Biosciences, Oxford, UK), progenitor cell markers (sex determining region Y-box 2, SOX2, 1 : 800; Millipore, Watford, UK) and paired box gene 6 (PAX6, 1 : 100; Santa Cruz Biotechnology, Santa Cruz, CA, USA) were applied overnight at 4 °C, and Dako Envision DAB (Dako, Cambridge, UK) was used as the chromogen to visualize nuclear labelling. Sections were double-labelled with monoclonal or polyclonal antibodies against glial markers (glial fibrillary acidic protein, GFAP, 1 : 1500; Dako, Cambridge, UK, and nestin, 1 : 6000; Millipore), or the microglial marker (ionized calcium binding adaptor molecule 1, IBA1, 1 : 1000; Dako), for 1 h at room temperature, and cytoplasmic immunolabelling was visualized with the Vector ImmPress VIP system (Vector Labs, Peterborough, UK). This generated seven sections per case: IBA1/MCM2, GFAP/MCM2, nestin/MCM2, GFAP/PAX6, GFAP/SOX2, nestin/PAX6 and nestin/SOX2.

For triple-labelled immunofluorescence, sections were incubated overnight in solutions consisting of monoclonals to combinations of MCM2, PAX6, GFAP and immature and mature neuronal cell markers, namely beta-III-tubulin (Sigma Aldrich, Poole, UK), neurofilament (SMI31; Sternberger Monoclonals, Lutherville, MD, USA), Doublecortin (Cell Signaling, Hitchin, UK), NeuN (Chemicon, Temecula, CA, USA) and polyclonal anti-nestin primary antibodies. In negative controls, one or both primary antibodies were omitted. Species-specific fluorophore-conjugated antibodies (Alexa Fluor 488 and 546; 1 : 100; Invitrogen, Paisley, UK) were applied for 3 h at room temperature. Sections were coverslipped using Vectashield DAPI mounting media (Vector Labs). Images were visualized and acquired using a Zeiss LSM700 confocal microscope (Zeiss, Oberkochen, Germany).

### Quantitative analysis

**Figure 1:**
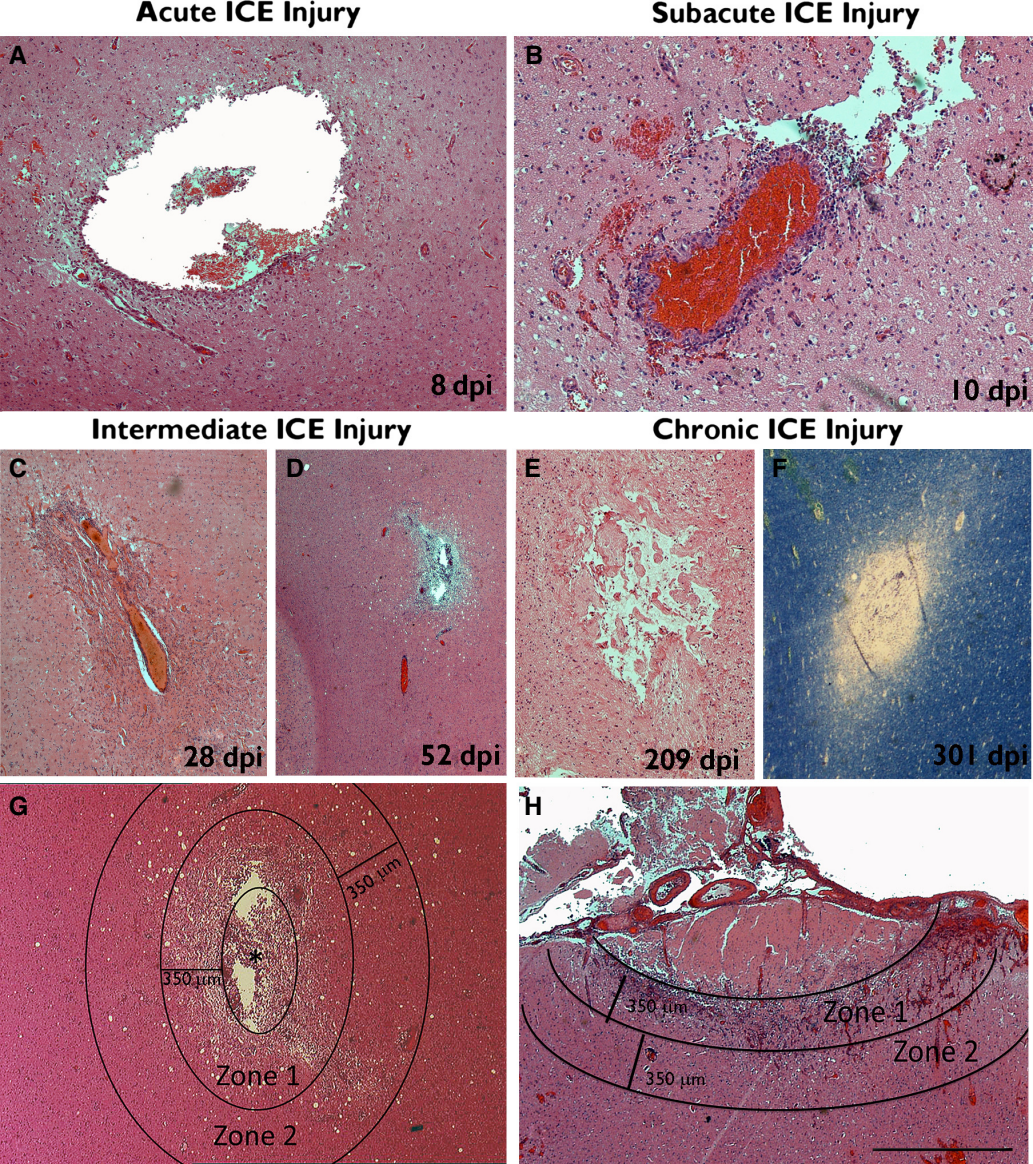
Appearance of ICE injuries of various ages and stages of organization. [All H&E-stained sections except F, which is a luxol–fast blue myelin preparation.] Small cavities from needle tracks are shown in the white matter at (A) 8 dpi, (B) 10 dpi, (C) 28 dpi, (D) 52 dpi, (E) 209 dpi and (F) 301 dpi, which were classified as acute, subacute, intermediate or chronic ICE injuries (see text). Inflammatory cell infiltrates are present in the acute–subacute ICE lesions but are sparse in the more chronic injuries. (G) Subacute ICE lesion (* marks the centre of the injury), annotated to illustrate the approximate positions of the zones evaluated. Zone 1 represented the immediate vicinity of the ICE injury, surrounding the cavity; the necrotic cell debris within the cavity of the ICE was not included in the analysis, and only viable, cellular tissue was selected. Zone 2 was of equal width and surrounding zone 1, corresponding to the penumbra of the ICE cavity. (H) Subacute ICE injury from a superficial grid placement indicating zones 1 and 2. Zone 1 in this case included the reactive granulation tissue in the margins of the lesion, extending from the subpial layer, but not including the non-viable, acellular coagulative necrosis in the central region. Scale bar in bottom right is equivalent to approximately 500 μm in C–F, 250 μm in A and B, and 700 μm in G and H. dpi, days post injury or implantation of the depth/grid electrode until resective surgery.

Immunolabelled sections were quantified using an image analysis program (Histometrix, Kinetic Imaging, UK) and Zeiss Axioskop microscope. Regions of interest were drawn at ×2.5 magnification in each case, using the H&E section as a reference (Fig.1). Zone 1 included the area immediately surrounding the viable edge of the ICE injury, to a width of 350 μm. The fibrin and necrotic cell debris in the centre of the ICE injuries was not included in zone 1, thus creating a doughnut shape for analysis around the electrode tracts and segment shape for superficial grid injuries (Fig.1G and H). Zone 2 was drawn with an equivalent width of 350 μm, to surround zone 1. These shapes and areas varied between cases, according to the contours of the ICE injury. The drawing template of these zones was saved and applied to each immunolabelled section from the same case. A further zone 3 was analysed, as a ‘control’ region, remote from the ICE injury site (≥2000 μm), within histologically normal-appearing tissue in the same tissue section. Within these three zones, immunopositive cells (excluding any vascular labelling) and double-labelled cells were counted at a high magnification (×40) by one researcher (J.G.). For zone 1 the area sampling fraction at ×40 was 100% with an equivalent area of randomly sampled fields analysed from zone 2. The sampled area for analysis in zones 1 and 2 ranged from 0.4 to 3.4 mm^2^ between cases [average 2 mm^2^, equivalent to 70 (×40 objective) high-power fields]. For zone 3, an area of 0.82 mm^2^ was sampled in each case [equivalent to 36 (×40 objective) high-power fields], which gave reproducible data on repeat counts. Mean cell densities per unit area for each marker, as well as the percentage of double-labelled cells in each zone for each marker were calculated. Cell counts in a proportion of cases were repeated to confirm reproducibility in the counting data.

The co-expression of nestin and GFAP in zone 1 was also investigated in four epilepsy cases with range of different ages of ICE injury (cases 5, 11, 16 and 17; 10, 28, 232 and 301 days respectively) using triple-labelled immunofluorescence. The ICE injury sites on nestin and GFAP co-labelled sections were tiled at ×20 objective on the Zeiss Axio Imager Z2 motorized fluorescence microscope. Zone 1 was drawn as described above for immunohistochemistry, and analysed with image pro plus version 6.3 (Media Cybernetics, Bethesda, MD, USA) for the quantification of the degree of colocalization. The image analysis program uses the Pearson coefficient as a measure of the degree of colocalization, whereby 1 indicates perfect overlap of signal, −1 indicates perfect negative correction and 0 indicates no correlation at all (Adler & Parmryd, 2010).

Statistical analysis was carried out using spss for windows (IBM corporation, version 21), and graphpad prism software (version 6.1) was used for the graphical representation of the data. Statistical methods used included non-linear regression analysis for the variation of cell densities with age of ICE injury (models selected for the goodness of fit and overall *P* value), non-parametric tests for comparison of values between zones, and multivariate analysis to investigate the influence of the underlying pathology and location of ICE injury (grey matter vs. white matter), with corrected exact *P* values shown.

## Results

### ICE injury ages and localization

Seventeen cases were included in the study with lesions ranging in age from 4 to 301 days post ICE injury (dpi) (Table1). These were grouped as acute injuries of a few days old (4–8 dpi; four cases), subacute injuries (10–13 dpi; six cases), intermediate injuries of several weeks age (28–70 dpi; four cases) and chronic injuries of over 6 months to 1 year (209–301 dpi; three cases) (Table1). The ICE injuries comprised penetrating needle-like track marks from depth electrode placement in 11 cases (Fig.1A–G) or crater-like infarcts of the superficial cortex following subdural grid placement in six cases (Fig.1H) with varying stages of organization at the margins of these injuries. ICE injuries were localized to the white matter in five (Fig.1D), hippocampus in one and cortex in 11 (Fig.1H). In nine cases the underlying diagnosis was focal cortical dysplasia (FCD) and in two of these the ICE injury was in the vicinity of cortical dysplasia (cases 1 and 3). In all other cases, the ICE injury was in otherwise normal-appearing, non-dysplastic cortex. Despite this apparent heterogeneity of tissue samples, an initial comparison of all cell density measures (using multivariate analysis to account for age of lesion) showed no differences between FCD and non-FCD cases. The only difference was for IBA1 cell densities between white matter and cortical ICE injuries, with higher IBA1 densities in the cortex (*P* = 0.02). We also did not observe any significant difference in glial cell densities in the four patients with clinical complications of local wound or surface infections (treated with antibiotics) following ICE insertion, compared with those with an uncomplicated post–operative course (Table1; cases 7, 11, 14 and 15). These observations justified inclusion of all cases to evaluate gliosis over time.

### Inflammatory infiltrates and proliferation in ICE injuries

**Figure 2:**
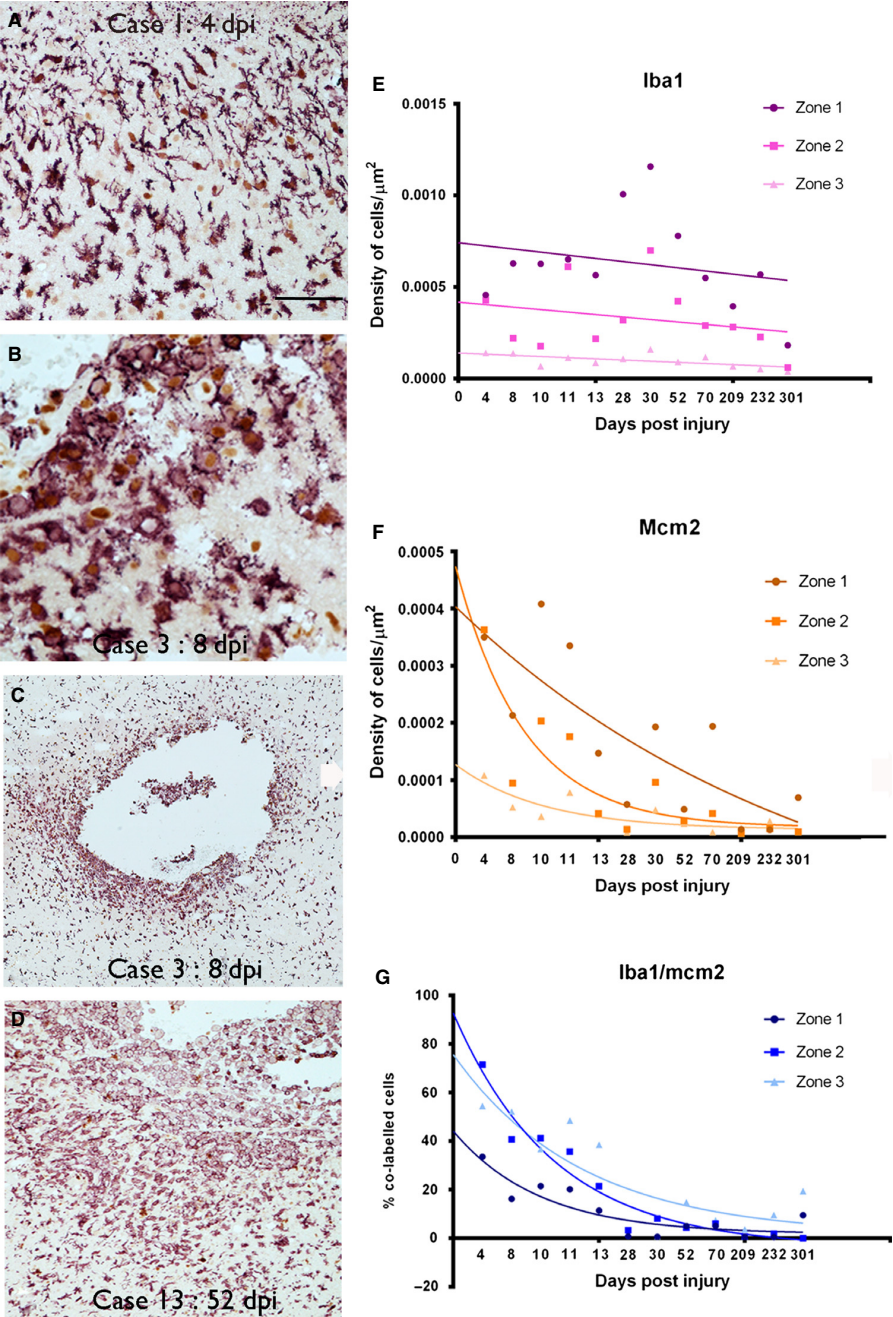
Proliferative and inflammatory activity with age of lesion. IBA1/MCM2 labelling (IBA1 in red and MCM2 nuclear stain in brown in A–D). In earliest ICE injuries cells with mainly microglial morphology were observed (A). Dense infiltrates of macrophages surrounding the ICE track cavity by q week (B and C) with a high proportion of these macrophages showing nuclear positivity (brown DAB labelling) being MCM2-positive (B). The cell infiltrates were still numerous at the edge of an ICE injury at 52 dpi (D). [For comparison, the identical region to D is shown with nestin/MCM2 in Fig.3B and GFAP/MCM2 in Fig.5B] (E). Graphical representation of the density of IBA1-positive microglial and macrophages cells in ICE injuries plotted against dpi. The data are shown for the three zones (1–3) of increasing distance away from ICE injury site (see text for details of zones) and each point represents the mean values from all sections and cases at each dpi time point with best fit non-linear regression analysis lines drawn in for each zone. The *x*-axis displays each time point of analysis and is a non-linear scale. Statistical analysis confirmed significantly greater densities in zone 1 than zones 2 or 3 (both *P* < 0.0001) with peak cell densities observed between 28 and 52 dpi. (F) A similar graph representing the density of MCM2-positive cells in ICE injuries plotted against dpi, displayed as each time point of analysis. There were significantly higher cell densities in zone 1 than zones 2 or 3 (both *P* < 0.001). Statistical non-linear regression analysis of data of MCM2 densities over time was significant in all three zones (zone 1: *P* = 0.0001, *R*^2^ = 0.463; zone 2: *P* = 0.0001, *R*^2^ = 0.417; zone 3: *P* = 0.0001, *R*^2^ = 0.283). (G) Graphical representation of the decline in the percentage of IBA1 cells colocalized with MCM2 with dpi shown in all three zones. The *x-*axis displays each time point of analysis and is a non-linear scale. A non-linear regression analysis of IBA1/MCM2 densities over time was significant in all three zones (zone 1: *P* = 0.001, *R*^2^ = 0.549; zone 2: *P* = 0.001, *R*^2^ = 0.723; zone 3: *P* = 0.0001, *R*^2^ = 0.548). dpi, days post injury or implantation of the depth/grid electrode until surgical resection. Scale bar in A is equivalent to approximately 85 μm, 40 μm in B, 120 μm in C and 250 μm in D.

IBA1 staining highlighted microglial activation and macrophage influx in the immediate vicinity of the ICE injury (Fig.2A–D). In addition, lower levels of widespread microglial up-regulation, including focal aggregates or nodules of microglia, were noted in the adjacent cortex and white matter. At the injury site, the morphology was predominantly microglial in early lesions (4 dpi; Fig.2A) with macrophages predominating by 8 dpi (Fig.2B and C). Quantitative analysis confirmed significantly greater densities of all IBA1-labelled cells in zone 1 than both zone 2 or zone 3 (both *P* < 0.0001) (Fig.2E). They were the predominant cell type in zone 1 in acute to chronic ICE injuries (Fig.2D), with an overall trend for regression with age of lesion in zone 1 (*P* = 0.054; *R*^2^ = 0.226).

We used MCM2 as a specific and sensitive cell-cycle marker, with nuclear labelling of cells licensed for replication (Freeman *et al*., 1999). As anticipated, we identified a significant decline in cell replicative activity in the ICE injuries with dpi, with declining MCM2 cell labelling in zone 1 (*P* < 0.001; Fig.2F). There were significantly higher densities of MCM2 cells in zone 1 than zone 2 (*P* < 0.0001) and in zone 1 than in zone 3 (*P* < 0.0001). A significant decline in proliferative capacity over time was also observed in zone 2 as well as in zone 3, remote from the ICE injury site (*P* < 0.001; Fig.2F). IBA1/MCM2 co-expressing cells (Fig.2G) were numerically the predominant replicating cell type in zone 1 in acute ICE injuries. There was a significant decline in the percentage of IBA1 cells that co-expressed MCM2 with increasing age of injury in zone 1 (*P* = 0.001) as well as in zone 2 (*P* = 0.001) and zone 3 (*P* < 0.0001; Fig.2G).

### Nestin-expressing cells

**Figure 3:**
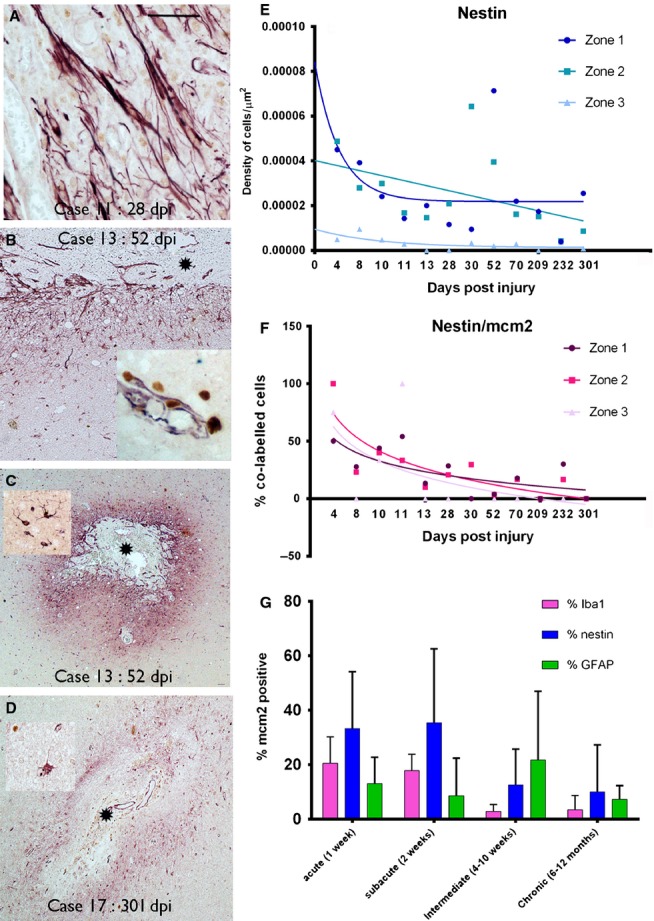
Nestin-expressing cells (nestin cells labelled in red and MCM2 nuclear labelling in brown in A–D). (A) ‘Strap-like’ or bipolar, elongated NECs were a prominent morphological cell type in the early ICE lesions in zone 1 as shown in a 28-day-old injury. (B) The gradient of distribution of nestin in relation to the injury core (*) with elongated cells concentrating in the immediate zone and fewer, scattered multipolar cells away from the lesion (bottom of image); inset: elongated cells extending alongside new vessels into the core of the injury with a ‘double layer’ of nestin-positive NECs surrounding nestin-positive endothelium. (C) Nestin is strikingly concentrated around the immediate injury zone at low magnification; inset: a typical nestin-positive multipolar glial cell. (D) Nestin expression remained localized to the ICE, demarcating the injury site, in the most chronic lesions, as shown at 301 days; in older lesions degenerate-appearing NECs were visible with cytoplasmic vacuolation. (E) Graphical representation of the density of nestin-positive cells in ICE injuries plotted against dpi. The data are shown for the three zones (1–3) of increasing distance away from the ICE injury site (see text for details of zones) with each point representing the mean values from all sections and cases analysed at each dpi time point; best fit non-linear regression analysis lines are shown for each zone. The *x-*axis displays each time point of analysis and is a non-linear scale. Statistical analysis showed no significant difference in cell densities between zone 1 and 2 but significantly lower cell densities in zone 3 compared with zone 1 (*P* < 0.0001). There was a significant regression in NEC density with dpi in zone 2 (*P* < 0.0001, *R*^2^ = 0.233). (F) A similar graphical representation for the decline in the percentage of nestin cells colocalized with MCM2 with dpi is shown for all three zones. Statistical non-linear regression analysis of data showed a significant decline in this index for nestin in zones 1 and 2 but not zone 3 (zone 1: *P* = 0.038, *R*^2^ = 0.257; zone 2: *P* = 0.024, *R*^2^ = 0.295; zone 3: *P* = 0.138, *R*^2^ = 0.14). (G) Bar chart of the percentages of glial cell types colocalizing with MCM2 indicating their regenerative index; the data are shown for zone 1 (the region immediately surrounding the ICE injury) and for acute, subacute, intermediate and chronic ICE injuries (see text for definition of intervals). This illustrates the decline in the MCM2 index for IBA1 and nestin populations with chronicity of injury whereas a similar decline is not seen for GFAP-positive glia. NECs in acute and subacute lesions have the highest replicative index. dpi, days post injury or implantation of the depth/grid electrode until resective surgery. Asterisk in each figure indicates the core or centre of the ICE injury. Scale bar is equivalent to approximately 40 μm in A, and 250 μm in B–D.

Qualitative evaluation of nestin-expressing cells (NECs) over this time series of ICE injuries highlighted a population primarily associated with the immediate vicinity of the injury site. ‘Strap-like’ or bipolar elongated cells were present in the earliest lesions (from 4 dpi) around the injury site (Fig.3A). From 8 dpi, NECs and processes were interspersed around and between new capillaries at the organizing margin of the ICE injury (Fig.3B). Condensation of cell processes formed a boundary wall, demarcating the injury site (Fig.3B–D). By 28 dpi, in-growths of NECs and fibres into the ICE cavity, alongside new capillaries, were a more established feature, with bipolar NECs often surrounding the nestin-positive endothelial cells, forming a ‘double layer’ (Fig.3B; inset). More distal to the ICE injury, multipolar NECs with delicate branching processes predominated, not associated with vessels but in fewer numbers than in the immediate injury site (Fig.3C and D, inset). In the oldest ICE injuries, hypertrophic as well as degenerate NECs were seen, with dense granular–globular cytoplasmic inclusions (Fig.3D). A persistence of nestin-positive fibres remained in the oldest lesions, demarcating the injury site as a pauci-cellular ‘fibrous scar’ (Fig.3D).

Quantitative analysis confirmed that NECs were present in significantly greater density in zone 1 than zone 3 (*P* < 0.0001) in ICE injuries of all age, but there was no significant difference in densities between zones 1 and 2 (*P* = 0.5; Fig.3E). Peak NEC densities were observed in ICE injuries of around 52 days in zone 1 and 30 days in zone 2 and, although their numbers declined over time in all three zones, statistically there was only a significant decline of NEC density in zone 2 (*P* < 0.0001; Fig.3E). There was a significant regression in the percentage of MCM2-expressing NECs with dpi in both zones 1 (*P* = 0.038) and 2 (*P* = 0.024) (Fig.3F; *P* < 0.05). More than twice the fraction of NECs were also MCM2-expressing compared with GFAP cells in zone 1 in acute ICE lesions (Fig.3G), highlighting their predominant replicative activity in early stages of injury repair processes.

**Figure 4:**
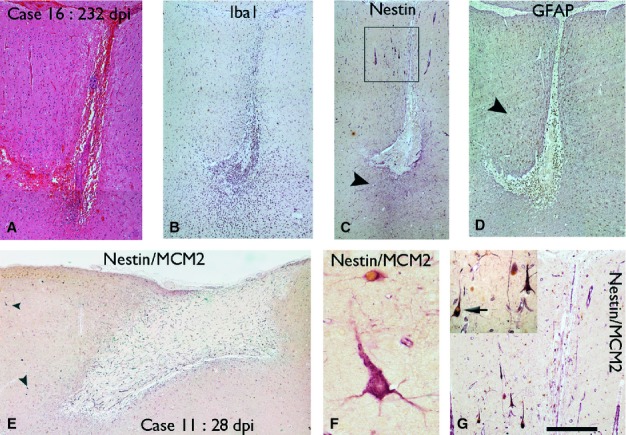
Nestin-expressing pyramidal cells at the margins of ICE injuries. A cortical injury of 232 days as visualised with H&E (A), and IBA1 showing macrophage infiltration (B). Nestin at low magnification highlights cells and processes mainly around the ICE injury (arrowhead) (C) whereas GFAP highlights a wider field of glial reaction in the surrounding tissue (D, arrowhead). (E) Nestin/MCM2 immunohistochemistry in a superficial grid injury of the cortex of 28 dpi showed scattered nestin-positive pyramidal cells in the marginal cortex (arrowheads); these are shown at higher magnification in F with nestin-expressing cells (labelled red) showing a striking pyramidal morphology and apical and basal dendrite-like processes. An adjacent small NEC (red) which also has nuclear MCM2 labelling (brown) is observed. (G) Similar nestin-positive pyramidal cells were seen at the 232 dpi with the region shown in the box in C enlarged and shown at higher magnification in the inset. The cells showed radial orientation of process and occasional cell (arrow) co-expressed nuclear MCM2. The scale bar in the lower right hand corner corresponds to approximately 500 μm in A–E, 120 μm in G and 35 in F.

**Figure 5:**
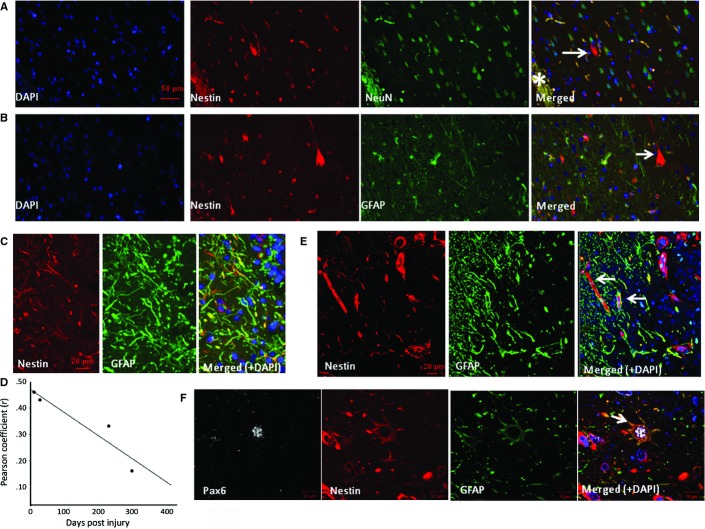
Characterization of NECs in zone 1. Double-labelling in case 16 (232 dpi) confirmed nestin-expressing pyramidal-like cells (arrow) but no co-expression of NeuN (A) or GFAP (B). (C) In case 5 (10 dpi), nestin-positive processes were observed in zone 1 with focal colocalization with GFAP. (D) The degree of colocalization between nestin and GFAP in four cases in zone 1 (cases 5, 11, 16 and 17 with injury of 10, 28, 232 and 301 days old, respectively) decreased with increasing dpi [*R*^2^ = 0.8822, Spearman correlation (*r*) = 0.939]. (E) Confocal image of zone 1 in case 3 (8 dpi) showed central localization of nestin filaments in some cells with peripheral arrangement of GFAP-positive filaments. (F) NECs expressing PAX6 and GFAP (arrow) were also observed in zone 1. *ICE central cavity on images. Scale bars = 50 μm (A and B), 20 μm (C and E) and 10 μm (F).

A further qualitative observation in two cases was occasional nestin-positive pyramidal shaped cells in the margins of intracortical ICE injures in non-dysplastic cortex (28–232 dpi; Fig.4A–E). These cells appeared polarized, bearing apical and basal dendrite-like processes (Fig.4F and G); these cells were occasionally MCM2-positive (Fig.4G, inset) but PAX6- and SOX2-negative (not shown). Further confocal microscopy confirmed these nestin-positive pyramidal cells did not stain for GFAP and also did not colocalize with mature neuronal markers, NeuN and neurofilament (SMI31; Fig.5A and B). Although co-expression of cells around the ICE with nestin and doublecortin or beta-III-tubulin was observed (not shown), it was not confirmed in the NECs with pyramidal morphology.

### GFAP-expressing glia

**Figure 6:**
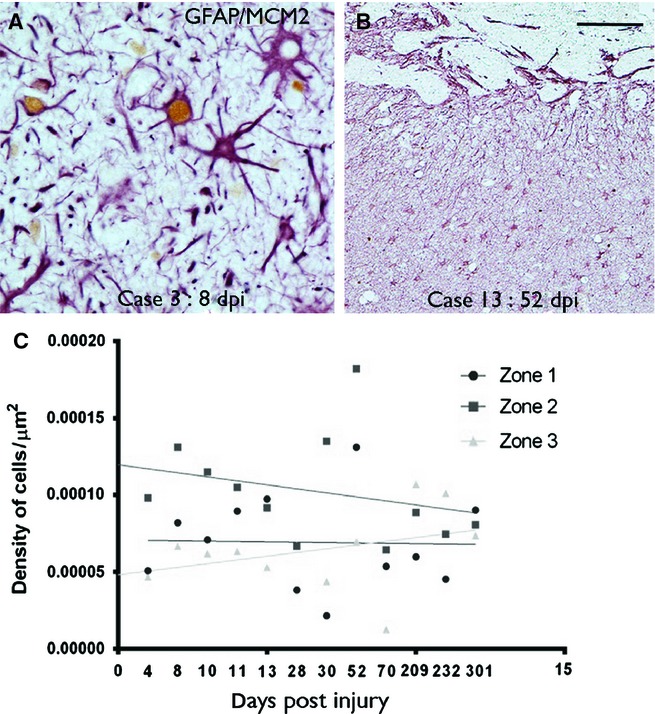
GFAP-expressing glia at injury site. (A) A proportion of multipolar, reactive and hypertrophic astrocytes (GFAP in red) in zone 1 in a 8-day-old lesion show nuclear labelling with MCM2 (brown nuclear labelling). (B) At low power, GFAP-positive astrocytes and processes showed a more even distribution in the tissue surrounding the ICE injury cavity edge, at the top of the figures (compare with the same region as shown with nestin in Fig.3B). (C) Graphical representation of the density of GFAP-positive cells in ICE injuries plotted against dpi. The data are shown for the three zones (1–3) of increasing distance away from the ICE injury site (see text for details of zones) and each point represents the mean values for all cases at that time point with regression lines drawn in for each zone. The *x*-axis displays each time point of analysis and is a non-linear scale. GFAP-positive cells showed highest densities in zone 2 in ICE injuries. Statistical non-linear regression analysis of data of GFAP cell densities with dpi was not significant for zones 1 and 2 with a trend for an increase in density in zone 3 (zone 1: *P* = 0.664, *R*^2^ = 0.004; zone 2: *P* = 0.118, *R*^2^ = 0.049; zone 3: *P* = 0.047, *R*^2^ = 0.078) and no significant differences for GFAP/MCM2 co-labelled cells [graph not displayed (zone 1: *P* = 0.66, *R*^2^ = 0.013; zone 2: *P* = 0.063, *R*^2^ = 0.212; zone 3: *P* = 0.83, *R*^2^ = 0.003)]. dpi, days post injury or implantation of the depth/grid electrode until resective surgery. Scale bar is equivalent to approximately 35 μm in A and 125 μm in B.

GFAP-positive reactive astrocytes were present from the earliest ICE injuries to oldest lesions. GFAP-positive astrocytes were identified in zone 3 control region from the earliest lesions, as a reflection of baseline levels of astrocytic gliosis, with a 10-fold higher density than NEC (4.7 × 10^−5^ vs. 4.8 × 10^−6^ μm^2^; Figs3E and 6C). Multipolar GFAP-positive reactive cells were there predominant morphological cell type (Fig.6A), both near the injury and extending into adjacent tissues, with a more even distribution than nestin [Figs6B (compare with nestin in Fig.3B), and 4C and D). In zone 1, bipolar GFAP-positive cells similar to nestin were noted in the immediate margins of the cavity (Fig.5C). Quantitative studies confirmed the highest densities of GFAP-positive cells in zone 2, with no significant difference in density of GFAP-positive cells between zones 1 and 3 (*P* = 0.38; Fig.2D). There was no significant regression in GFAP cell density with dpi in zone 1 or 2, and a trend for increased density with dpi in zone 3 (*P* = 0.047; Fig.2D). Unlike nestin, linear regression analysis showed no significant decline in the percentage of GFAP/MCM2 co-labelled cells with dpi in any of the three zones. Whereas the regenerative index of NECs (percentage of nestin-positive cells that co-labelled with MCM2) in zone 1 diminished by approximately two-thirds from acute to chronic lesions, the regenerative index for GFAP diminished by less than a half (Fig.3G). Co-expression of nestin and GFAP in zone 1 was observed with immunofluorescence studies (Fig.5C–F) particularly in the elongated cell processes. Some cells showed central nestin filaments with surrounding GFAP in glia cell processes at the organizing margin (Fig.5E). Quantitative analysis showed that extent of nestin/GFAP colocalization in zone 1 decreased with increasing dpi (*n* = 4, Pearson colocalization correlation 0.939; Fig.5D).

### PAX6 and SOX2 expression in ICE lesions

We used expression of PAX6 and SOX2 as fate-specification transcription factors to further explore different glial lineage and differentiation pathways. PAX6 is more commonly regarded as specifying a pro-neuronal fate during development (Klempin *et al*., 2012) as well as adult CNS (von Bohlen und Halbach, 2011; Tripathi & Mishra, 2012). However, lower levels of neuronal expression are noted in adult cortex (Duan *et al*., 2013) with evidence for PAX6 promotion of astrocytic differentiation (Sakurai & Osumi, 2008). SOX2 is a highly conserved transcription factor vital for maintaining neural stem cells and promoting pluripotency, promoting neuronal differentiation in astrocyte cultures (Boda & Buffo, 2010; Pevny & Nicolis, 2010; Driessens & Blanpain, 2011) and regarded as a marker of neurogenesis (von Bohlen und Halbach, 2011).

**Figure 7:**
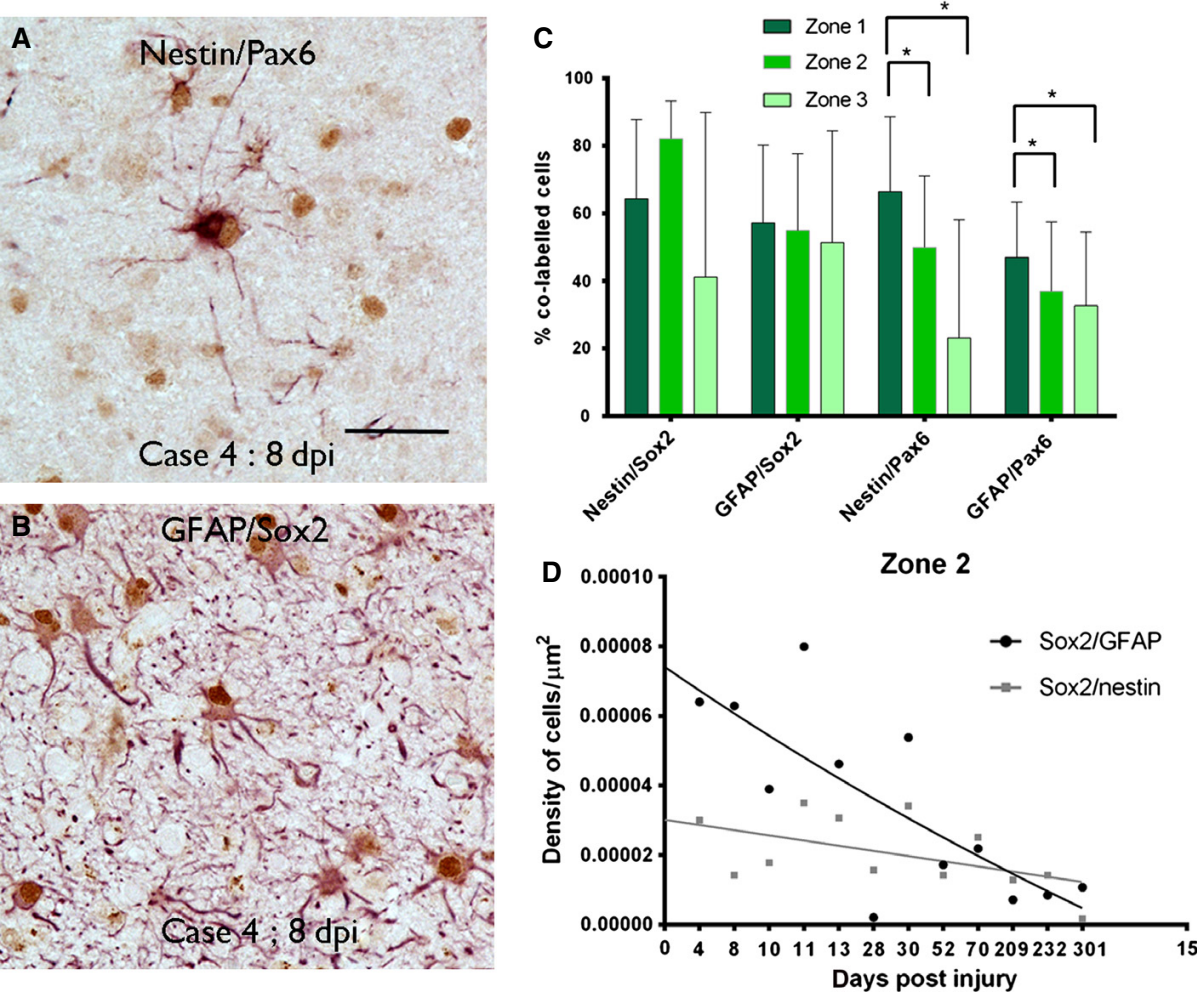
SOX2 and PAX6 expression at injury site. A proportion of multipolar, reactive and hypertrophic glial cells in the margins of the injury showed nuclear labelling (brown) with PAX6 and SOX2 as shown for nestin (in red)/PAX6 (A) and GFAP(red)/SOX2 (B). (C) Bar chart of the percentage of GFAP- or nestin-expressing cells that colocalized with SOX2 or PAX6 in the three zones is shown for all time points (error bars are SD). There was a significantly greater percentage (of both GFAP and nestin) PAX6-positive glial cells in zone 1 (immediately surrounding the injury) than in zone 2 or 3 in all cases (*P < 0.001). (D) Graphical representation of the density of SOX2/GFAP and SOX2/nestin co-labelled cells with dpi shown for zone 2. Each point represents the mean values for all cases at that time point with regression lines drawn in for each zone. The *x*-axis displays each time point of analysis and is a non-linear scale. Statistical non-linear regression analysis of data confirmed a significant decline in the percentage of SOX2/GFAP expressing cells with dpi (*P* = 0.053, *R*^2^ = 0.028) but not nestin/SOX2 cells (*P* = 0.053, *R*^2^ = 0.22). Scale bar in A is equivalent to approximately 40 μm.

PAX6 nuclear labelling of both NECs and GFAP-positive cells was seen in the vicinity of the ICE injury (Fig.7A). SOX2 nuclear labelling was observed in a high proportion of NECs and GFAP cells of all morphologies, both in the vicinity of the ICE injury and in distal cortex and white matter, and in lesions of all ages (Fig.7B). There were significantly more PAX6-positive glial cells in zone 1 than in zones 2 or 3 in all cases (*P* < 0.001; Fig.7C) with a significantly greater percentage of NECs co-expressing PAX6 than did GFAP-positive cells (*P* = 0.001). There were no significant differences in the percentage of SOX2-positive glia (NECs or GFAP-positive cells) between zones (*P* = 0.35; Fig.7C). There was no significant decline in the percentage of NECs or GFAP-positive cells that also labelled for PAX6 with dpi; there was a significant reduction in the percentage of GFAP-positive cells that co-expressed SOX2 in zone 2 only with dpi (*P* = 0.031) but not for nestin-SOX2 (Fig.7D).

## Discussion

We have utilized ICE injuries encountered in epilepsy surgical resections as a model to study the spatio-temporal progression of gliosis/gliogenesis in human CNS repair. The key findings of the present study were the identification of NECs as spatially associated with the injury site, intimately related to new vessels in the organization process, with NEC numbers peaking following the height of microglial recruitment. We demonstrate that NECs represent a dynamic population, shown to be the most proliferative cell type in acute injuries with a wide range of cell morphologies and evidence for astrocytic differentiation. NECs declined in number in older injuries, with evidence of cellular degeneration, but with some persistence of nestin expression demarcating the residual cortical scar. ICE injuries are analogous to experimental CNS injury models (Frisen *et al*., 1995; Krum & Rosenstein, 1999; Shibuya *et al*., 2002; Baumgart *et al*., 2012; Suzuki *et al*., 2012; Wanner *et al*., 2013), but enable the study of the greater complexity of glial cell reactions in the human brain (Oberheim *et al*., 2009). ICE injuries in surgical tissues have advantages as a model of CNS repair processes over the study of pathologies, as brain trauma or stroke, because well-defined lesions of a precise age are visualized in optimally preserved tissue without superimposed secondary effects relating to ongoing cerebral ischaemia, brain swelling or natural disease progression. In our current series, statistical confirmation of the anticipated spatio-temporal decline in overall cell regeneration and microglial/macrophage influx supports to the validity of this model and our evaluation methods.

In a previous cortical photo-injury experiment, a time-course study of nestin demonstrated expression at 4 days, peaking at 7 days and becoming undetectable by 30 days following injury (Suzuki *et al*., 2012), paralleling observations in other experimental injury models (Frisen *et al*., 1995; Krum & Rosenstein, 1999; Shibuya *et al*., 2002; Suzuki *et al*., 2012). We also noted NECs in the earliest ICE injuries at 4 days, but their density in our human tissue samples peaked in lesions of 30–50 days old, with a marginally earlier increase in zone 2 than in zone 1. NECs were more often MCM2- or PAX6-positive than GFAP-positive glia studied but with a significant regression of their regenerative activity over time, particularly after 28 weeks. The source of NECs in experimental injuries has been debated regarding their local proliferation vs. recruitment into the injury site, and also from differentiated glia or residual pluripotent less differentiated cells. For example, studies of spinal cord injuries have proposed that NECs are recruited to the necrotic core from subpial or ependymal GFAP-positive glia (Frisen *et al*., 1995; Shibuya *et al*., 2002). Genetic fate mapping studies have confirmed that differentiated resting astrocytes can proliferate following injury with a subset expressing nestin (Buffo *et al*., 2008), supporting a capacity for astroglia to de-differentiate.

In our series we observed a close spatial relationship of NECs with the newly formed vessels surrounding the organizing ICE injury site. The proximity of NECs to vessels has previously been noted in acute experimental stab wound injuries (Krum & Rosenstein, 1999), with elongated NECs proposed to migrate into the lesion core along vessels (Suzuki *et al*., 2012). Morphological studies of NECs in other experimental injury models also comment on ‘elongated cells with slender processes’ akin to migrating cells (Frisen *et al*., 1995; Krum & Rosenstein, 1999; Wanner *et al*., 2013) and NECs cells with bipolar morphology, likened to radial glia, have also been noted human tissues in active multiple sclerosis plaques (Moreels *et al*., 2008). Similar ‘strap-like’ elongated cells were prominent in the margins of the earliest ICE lesions of our series, in proximity to the new capillaries in the region of organization, reminiscent of pericytes. It has been proposed that one role of NECs is to form a scaffold-like framework, supporting angiogenesis. A recent study in a rat model of ischaemic stroke also found NECs in the perivascular spaces of the organizing lesion, distinct from nestin-expressing endothelial cells (Shin *et al*., 2013) with speculation that NECs might arise from pericyte/precursor type cells nurtured in the perivascular niche. In human autopsy material from patients with strokes, small NECs were also reported in the vicinity of vessels; these cells were more frequently observed in 4-day-old strokes and declining in number after day 30 (Nakayama *et al*., 2010). It is recognized that nestin expression can be induced by the epidermal growth factor (EGF) signalling pathway and extracellular molecules including thrombin and factors from vascular smooth muscle cells (Hyder *et al*., 2011). In addition, vascular endothelial growth factor (VEGF) expression and VEGFR have been shown in NECs in acute experimental stab wounds (Krum & Rosenstein, 1999). Mobilization and differentiation of bone-marrow-derived stem cells may represent an alternative source of NECs at the site of CNS injury with blood–brain barrier breakdown (Borlongan *et al*., 2011). NECs in early injuries have also been shown to share some characteristics of macrophages (Shin *et al*., 2013). In human multiple sclerosis tissue, it was noted than NECs were in close proximity to microglia, and that TGF-β1 expression by microglia upregulated nestin expression (Moreels *et al*., 2008). In the current study the similar time course in the peak densities of IBA1-positive microglia and NECs could support a relationship between these cell types. These converging data suggest an interplay between the molecular signals regulating angiogenesis, microglial responses and generation of NECs (Font *et al*., 2010) in the vicinity of brain injuries, which invites further study.

There are also data from experimental and human tissues regarding the persistence of an NEC pool in the normal adult mammalian brain as a potential source for glial regeneration (Moreels *et al*., 2008; Hendrickson *et al*., 2011). A recent study identified and categorized adult mammalian NECs based on their localization and morphology (Hendrickson *et al*., 2011): class I NECs had small soma and three to four branching processes, class II NECs resided in the ventricular zone, class III NECs represented rare NeuN+ cells in layer II of the pyriform cortex and pyramidal cell layer of the hippocampus and class IV NECs were widely distributed small multipolar cells, often in a satellite location in relation to neurons (Hendrickson *et al*., 2011). Type I NECs in experimental models have been shown to proliferate 20-fold in response to injury (Hendrickson *et al*., 2011). In our present series, small, multipolar NECs were also observed in regions away from the immediate injury site, corresponding morphologically to these type I cells of Hendrickson, in support of a resident ‘latent’ population. It is plausible that these persistent NECs in the mature adult brain act as the pool for the cell proliferation at ICE acute injury sites. Although we did observe earlier peaks in NECs in zone 2 than 1, it is not possible with the current study design and small number of cases to draw conclusions regarding any local influx or migration of NECs into the acutely injured region.

As distinct from NECs, GFAP-positive astroglia showed no significant differences in their densities between zones or decline in regenerative potential with injury age. The phenotype of GFAP-positive cells was similar to that recently described in experimental injuries, with elongated forms near the acute injury site and stellate cells distally (Wanner *et al*., 2013), and no decline in their number was noted from day 7 to 30 in another study (Suzuki *et al*., 2012). Double-labelling studies in ICE injuries of different ages in the present series confirmed co-expression of GFAP and nestin in a subset of cells and elongated processes in zone 1, which declined with increasing age of lesion. Co-expression studies in experimental injuries at different stages have also identified populations of glial cells, including subsets of GFAP−/nestin+, GFAP+/nestin+ and GFAP+/nestin− cells (Shibuya *et al*., 2002; Shimada *et al*., 2012; Suzuki *et al*., 2012; Wanner *et al*., 2013), with nestin+/GFAP+ co-expressing populations declining significantly with lesion age, as in our series. One interpretation of these observations is that there are two different processes in repair: astrocytic differentiation in proliferating NECs at injury sites (comparable to developmental gliogenesis) and lower rates of proliferation of more remote, stellate, differentiated GFAP+ astrocytes (gliosis). It is likely that distinct populations of glia, with spatiotemporal diversity in relation to injury site, are involved in CNS repair.

We utilized expression of PAX6 and SOX2 as fate-specification transcription factors to further delineate different glial lineage and differentiation pathways. We observed that a significantly greater proportion of NECs co-labelled with PAX6, with a proportion of GFAP-positive glia also PAX6-positive. PAX6 expression in astroglial cell lines (with regulatory roles in differentiation and proliferation) has recently been established (Sakurai & Osumi, 2008; von Bohlen und Halbach, 2011; Gomez-Lopez *et al*., 2011). PAX6-positive cells, although identified through the cortex, were concentrated at the lesion site. It is tempting to speculate that GFAP/PAX6 co-expressing cells (which predominated in zone 1) represent glial-differentiating NECs at the injury site, which invites further investigation. SOX2 is a highly conserved transcription factor, promoting neuronal differentiation (Boda & Buffo, 2010; Pevny & Nicolis, 2010; Driessens & Blanpain, 2011) and is upregulated adjacent to experimental injuries (Shimada *et al*., 2012; Wanner *et al*., 2013). In the present study, SOX2 was noted in both NEC and GFAP glial populations in equal proportion and through all zones of the injuries, although there was evidence for regression over time, supporting its induction in brain repair processes.

In the margins of older ICE cases, we identified occasional NECs with striking pyramidal cell morphology. We lack definite evidence that they represent fully differentiated neurons as they did not express mature neuronal markers including NeuN. The earliest time point they were noted was in 4-week injuries and were always in close proximity to the injury site and in cortex without evidence of cortical dysplasia. Nestin/NeuN co-expressing cells, occasionally reported in normal mammalian cortex, have been hypothesized to represent a subset of neurons retaining a degree of plasticity (Hendrickson *et al*., 2011). Nestin expression in mature neurons may also signify initiation of a neuronal cell death pathway, either prior to cell cycle re-entry or as a transient neuroprotective strategy to stabilize microtubules. Therefore, one explanation is that nestin expression in pyramidal-like cells near to ICE injuries represents degenerative cortical neurons. Against this, preliminary investigations did not confirm cell colocalization with neurodegenerative proteins (such as phosphorylated tau – data not shown). An alternative explanation is that nestin-positive pyramidal cells represent aberrant neurogenesis following incomplete differentiation of NECs in the later stages of cortical injury repair. Experimental cortical injury models have identified newly generated NeuN-positive neurons during the reparative process (Baumgart *et al*., 2012; Suzuki *et al*., 2012) and genetic fate mapping studies have also confirmed that, following injury, proliferating astroglia have a capacity for neuronal differentiation (Buffo *et al*., 2008).

Residual nestin labelling remained in the most chronic injuries in the series, of up to 301 days in age, forming the ‘fibrous’ pauci-cellular glial scar demarcating the injury site. In experimental CNS injuries, long-term persistence of nestin expression is a more variable finding (Krum & Rosenstein, 1999) although reported in lesions of up to 13 months old in one study (Frisen *et al*., 1995). A barrier role of nestin-positive fibres in ‘walling off’ the injury site to diminish ongoing diffusion of potentially damaging proteins has been proposed, with a reduction in the diffusion of IgG beyond the glial scar noted at 7 dpi in one model (Suzuki *et al*., 2012). NECs have recently been shown to have fibroblast-like properties in experimental infarcts, facilitating local structural remodelling (Shin *et al*., 2013). The potential local detrimental consequence of a compact glial scar on the restoration of local connections and function is a key issue in regenerative neuroscience. The prominent role of NECs as ‘scar-forming’ glia that we have observed in human tissues deserves further study with a view to potential therapeutic manipulation.

Our study demonstrates that ICE injuries provide a useful model to study CNS repair. The inherent limitations of using human tissue samples is that patients, by definition, have had drug-resistant epilepsy with pre-interventional gliosis (supported by our quantitative evaluations in control regions) as a baseline level on which these acute injuries are superimposed. Also, some electrodes may be positioned in more electrically active zones than others, which could also influence relative levels of gliosis. In addition, in some patients the electrodes are surgically removed at an interval prior to the tissue resection; it is possible that this procedure could further irritate the injury site. There was no observable influence of post-operative complications/infections in our small series on glial cell densities. The ICE injuries are small, limiting extensive histological studies in single cases. The injuries are also more unpredictably located in either grey or white matter and in normal vs. pathological tissue compared with controlled experimental conditions. We did not, however, identify significant differences in astrocyte numbers between pathology types or white vs. grey matter injuries [as also noted in one experimental study (Wanner *et al*., 2013)]; it is possible that our small sample size failed to identify statistically significant differences. Nevertheless, this study has confirmed NECs to be a dynamic cell population in neocortical injuries, ultimately contributing to the formation of the glial scar but with evidence to suggest a capacity for both astroglial and possible neuronal differentiation in human brain repair processes.

## Abbreviations

CNS: central nervous system
dpi: days post injury or implantation of the depth/grid electrode until resective surgery
FCD: focal cortical dysplasia
GFAP: glial fibrillary acidic protein
IBA1: ionized calcium binding adaptor molecule 1
ICE: intracranial electrode injuries
MCM: mini chromosome maintenance protein
NECs: nestin expressing cells
PAX6: paired box gene 6
SOX2: SRY (sex determining region Y)-box 2

## References

[b1] Adler J, Parmryd I (2010). Quantifying colocalization by correlation: the Pearson correlation coefficient is superior to the Mander's overlap coefficient. Cytom. Part A.

[b2] Baumgart EV, Barbosa JS, Bally-Cuif L, Gotz M, Ninkovic J (2012). Stab wound injury of the zebrafish telencephalon: a model for comparative analysis of reactive gliosis. Glia.

[b3] Blanc F, Martinian L, Liagkouras I, Catarino C, Sisodiya SM, Thom M (2011). Investigation of widespread neocortical pathology associated with hippocampal sclerosis in epilepsy: a postmortem study. Epilepsia.

[b4] Boda E, Buffo A (2010). Glial cells in non-germinal territories: insights into their stem/progenitor properties in the intact and injured nervous tissue. Arch. Ital. Biol.

[b5] von Bohlen und Halbach O (2011). Immunohistological markers for proliferative events, gliogenesis, and neurogenesis within the adult hippocampus. Cell Tissue Res.

[b6] Borlongan CV, Glover LE, Tajiri N, Kaneko Y, Freeman TB (2011). The great migration of bone marrow-derived stem cells toward the ischemic brain: therapeutic implications for stroke and other neurological disorders. Prog. Neurobiol.

[b7] Buffo A, Rite I, Tripathi P, Lepier A, Colak D, Horn AP, Mori T, Gotz M (2008). Origin and progeny of reactive gliosis: a source of multipotent cells in the injured brain. Proc. Natl. Acad. Sci. USA.

[b8] Chaboub LS, Deneen B (2012). Developmental origins of astrocyte heterogeneity: the final frontier of CNS development. Dev. Neurosci.

[b9] Driessens G, Blanpain C (2011). Long live sox2: sox2 lasts a lifetime. Cell Stem Cell.

[b10] Duan D, Fu Y, Paxinos G, Watson C (2013). Spatiotemporal expression patterns of Pax6 in the brain of embryonic, newborn, and adult mice. Brain Struct. Funct.

[b11] Font MA, Arboix A, Krupinski J (2010). Angiogenesis, neurogenesis and neuroplasticity in ischemic stroke. Curr. Cardiol. Rev.

[b12] Freeman A, Morris LS, Mills AD, Stoeber K, Laskey RA, Williams GH, Coleman N (1999). Minichromosome maintenance proteins as biological markers of dysplasia and malignancy. Clin. Cancer Res.

[b13] Frisen J, Johansson CB, Torok C, Risling M, Lendahl U (1995). Rapid, widespread, and longlasting induction of nestin contributes to the generation of glial scar tissue after CNS injury. J. Cell Biol.

[b14] Gomez-Lopez S, Wiskow O, Favaro R, Nicolis SK, Price DJ, Pollard SM, Smith A (2011). Sox2 and Pax6 maintain the proliferative and developmental potential of gliogenic neural stem cells *in vitro*. Glia.

[b15] Heinemann U, Kaufer D, Friedman A (2012). Blood-brain barrier dysfunction, TGFbeta signaling, and astrocyte dysfunction in epilepsy. Glia.

[b16] Hendrickson ML, Rao AJ, Demerdash ON, Kalil RE (2011). Expression of nestin by neural cells in the adult rat and human brain. PLoS One.

[b17] Hyder CL, Isoniemi KO, Torvaldson ES, Eriksson JE (2011). Insights into intermediate filament regulation from development to ageing. J. Cell Sci.

[b18] Kang W, Hebert JM (2011). Signaling pathways in reactive astrocytes, a genetic perspective. Mol. Neurobiol.

[b19] Klempin F, Marr RA, Peterson DA (2012). Modification of pax6 and olig2 expression in adult hippocampal neurogenesis selectively induces stem cell fate and alters both neuronal and glial populations. Stem Cells.

[b20] Kovacs R, Heinemann U, Steinhauser C (2012). Mechanisms underlying blood–brain barrier dysfunction in brain pathology and epileptogenesis: role of astroglia. Epilepsia.

[b21] Krum JM, Rosenstein JM (1999). Transient coexpression of nestin, GFAP, and vascular endothelial growth factor in mature reactive astroglia following neural grafting or brain wounds. Exp. Neurol.

[b22] Liu JY, Thom M, Catarino CB, Martinian L, Figarella-Branger D, Bartolomei F, Koepp M, Sisodiya SM (2012). Neuropathology of the blood–brain barrier and pharmaco-resistance in human epilepsy. Brain.

[b23] Love S, Louis DN, Ellison D, Greenfield JG (2008). Greenfield's Neuropathology.

[b24] Moreels M, Vandenabeele F, Dumont D, Robben J, Lambrichts I (2008). Alpha-smooth muscle actin (alpha-SMA) and nestin expression in reactive astrocytes in multiple sclerosis lesions: potential regulatory role of transforming growth factor-beta 1 (TGF-beta1). Neuropath. Appl. Neuro.

[b25] Nakayama D, Matsuyama T, Ishibashi-Ueda H, Nakagomi T, Kasahara Y, Hirose H, Kikuchi-Taura A, Stern DM, Mori H, Taguchi A (2010). Injury-induced neural stem/progenitor cells in post-stroke human cerebral cortex. Eur. J. Neurosci.

[b26] Oberheim NA, Takano T, Han X, He W, Lin JH, Wang F, Xu Q, Wyatt JD, Pilcher W, Ojemann JG, Ransom BR, Goldman SA, Nedergaard M (2009). Uniquely hominid features of adult human astrocytes. J. Neurosci.

[b27] Pevny LH, Nicolis SK (2010). Sox2 roles in neural stem cells. Int. J. Biochem. Cell Biol.

[b28] Sakurai K, Osumi N (2008). The neurogenesis-controlling factor, Pax6, inhibits proliferation and promotes maturation in murine astrocytes. J. Neurosci.

[b29] Shibuya S, Miyamoto O, Auer RN, Itano T, Mori S, Norimatsu H (2002). Embryonic intermediate filament, nestin, expression following traumatic spinal cord injury in adult rats. Neuroscience.

[b30] Shimada IS, LeComte MD, Granger JC, Quinlan NJ, Spees JL (2012). Self-renewal and differentiation of reactive astrocyte-derived neural stem/progenitor cells isolated from the cortical peri-infarct area after stroke. J. Neurosci.

[b31] Shin YJ, Kim HL, Park JM, Cho JM, Kim SY, Lee MY (2013). Characterization of nestin expression and vessel association in the ischemic core following focal cerebral ischemia in rats. Cell Tissue Res.

[b32] Sofroniew MV, Vinters HV (2010). Astrocytes: biology and pathology. Acta Neuropathol.

[b33] Steinhauser C, Seifert G, Bedner P (2012). Astrocyte dysfunction in temporal lobe epilepsy: K+ channels and gap junction coupling. Glia.

[b34] Suzuki T, Sakata H, Kato C, Connor JA, Morita M (2012). Astrocyte activation and wound healing in intact-skull mouse after focal brain injury. Eur. J. Neurosci.

[b35] Tripathi R, Mishra R (2012). Aging-associated modulation in the expression of Pax6 in mouse brain. Cell. Mol. Neurobiol.

[b36] Vezzani A, French J, Bartfai T, Baram TZ (2011). The role of inflammation in epilepsy. Nat. Rev. Neurol.

[b37] Wachter B, Schurger S, Rolinger J, von Ameln-Mayerhofer A, Berg D, Wagner HJ, Kueppers E (2010). Effect of 6-hydroxydopamine (6-OHDA) on proliferation of glial cells in the rat cortex and striatum: evidence for de-differentiation of resident astrocytes. Cell Tissue Res.

[b38] Wanner IB, Anderson MA, Song B, Levine J, Fernandez A, Gray-Thompson Z, Ao Y, Sofroniew MV (2013). Glial scar borders are formed by newly proliferated, elongated astrocytes that interact to corral inflammatory and fibrotic cells via STAT3-dependent mechanisms after spinal cord injury. J. Neurosci.

[b39] Yuan J, Chen Y, Hirsch E (2012). Intracranial electrodes in the presurgical evaluation of epilepsy. Neurol. Sci.

